# A Simulated Patient Protocol for Implementing Evidence-Based Practices in Health Care Delivery

**DOI:** 10.21203/rs.3.rs-3395246/v1

**Published:** 2023-11-14

**Authors:** Ellen Green, Megan Hamm, Catherine Gowl, Reed Van Deusen, Jane M. Liebschutz, J. Deanna Wilson, Jessica Merlin

**Affiliations:** Arizona State University; University of Pittsburgh School of Medicine; University of Pittsburgh School of Medicine; University of Pittsburgh School of Medicine; University of Pittsburgh School of Medicine; University of Pennsylvania Perelman School of Medicine; University of Pittsburgh School of Medicine

**Keywords:** Evidence-Based Practices (EBPs), Simulated Patient Methodology, Implementation Strategy Development

## Abstract

**Background:**

Substantial work has been done to update or create evidence-based practices (EBPs) in the changing health care landscape. However, the success of these EBPs is limited by low levels of clinician implementation. The goal of this study is to describe the use of simulated patient (SP) methodology as a framework to develop implementation bundles to increase the effectiveness, sustainability, and reproducibility of EBPs across health care clinicians. The primary outcome was identifying likely facilitators for the successful implementation of EBP. Our secondary outcome was the assess the feasibility of using SPs to illuminate likely implementation barriers and facilitators.

**Methods:**

We observed 12 primary care clinicians’ first-time experiences with six unique decision-making algorithms for use with patients exhibiting concerning behaviors associated with long-term opioid therapy (LTOT) for chronic pain over Zoom. Each clinician was paired with two simulated patients trained to portray individuals with one of the concerning behaviors addressed by the algorithms. The Standardized Patient-evaluations were followed by CFIR guided one-on-one interviews with the clinicians.

**Results:**

The SP portrayal illuminated factors that were pertinent to address in the implementation bundle. SPs were realistic in their portrayal of patients with concerning behaviors associated with LTOT for chronic pain, but clinicians also noted that their patients in practice may have been more aggressive about their treatment plan.

**Conclusions:**

SP simulation provides unique opportunities for obtaining crucial feedback to identify best practices in the adoption of new EBPs for high-risk patients.

## Background

Timely adoption of current evidence-based practices (EBPs) is key to ensuring high-quality care in our changing health care environment. Simply developing EBPs is not enough to guarantee that practices are implemented as is it can take decades for adoption to occur without well-designed implementation strategies.^1^ Evaluation of implementation strategies outside of an active practice setting increases the likelihood of dissemination, long-term adoption, and appropriate use of evidence-based practices (EBPs) by providing a controlled environment for assessment, feedback, and identification of barriers and facilitators, without the constraints and complexities of real-world clinical practice.^2–4^ To this end, simulated patient (SP) methodology is a valuable tool for developing implementation strategies for EPBs prior to their use in practice.

SPs are people trained to portray complex behaviors and react as an actual patient would to a clinician in real time creating a fully interactive patient-clinician experience outside of real-world practice.^5^ SPs can be trained to consistently exhibit specific emotions (e.g., anger^6^), desires (e.g., prescriptions), and/or patient needs (e.g., language barriers^7^) across clinicians. Simulation structure is flexible and can reflect either a single patient encounter or multiple patient visits portraying the passage of time depending on the application (e.g., teach providers how to perform a physical exam vs. re-evaluate patients after a new prescription). While SP methodology is commonly used to train and test clinicians on new techniques,^8–12^ this methodology is underutilized in the planning phases of implementation science. We contend that this way of working with SPs for the development of implementation strategies is a novel and important approach.

There are several advantages to using SP methodology as a part of an implementation strategy. First, the consistent portrayal of a patient case can help identify gaps in EBP implementation and facilitate targeted solutions for future implementation. Second, recruiting clinicians from different practices to use the EBPs with SPs can provide insight into how the EBP would work in their unique practice setting after first-hand experience. This provides richer insight than feedback from implementing an EBP into a singular practice that may not generalize to other clinics. Likewise, evaluating an EBP outside of the bustle of a typical clinical practice provides clinicians with immediate and protected time for debriefing. Without good feedback, it is difficult to identify areas of improvement for implementation. Also, developing implementation strategies for EBPs in practice can be high risk for patients. The use of SPs provides a safe environment to develop implementation strategies and gain active experience with EBPs without putting patients at risk. Lastly, SPs can provide insight into events that may be uncommon or take a long time to occur in practice, which can expedite necessary adaptation of implementation strategies for EBPs. Overall, SPs may provide a critical step in increasing the likelihood of a successful adoption of an EBP by identifying the barriers and facilitators prior to implementation in the field. In this article, we provide an example of using the SP methodology for a new EBP. We used observation and discussion from one-on-one structured interviews to develop an implementation bundle to increase the likelihood of effective, sustainable, and reproducible adoption in practice. Our approach was guided by the Consolidated Framework for Implementation Research (CFIR), a commonly used tool to guide qualitative inquiry about how clinicians would implement EBPs in practice.^13^

## Methods

**Figure 1:**
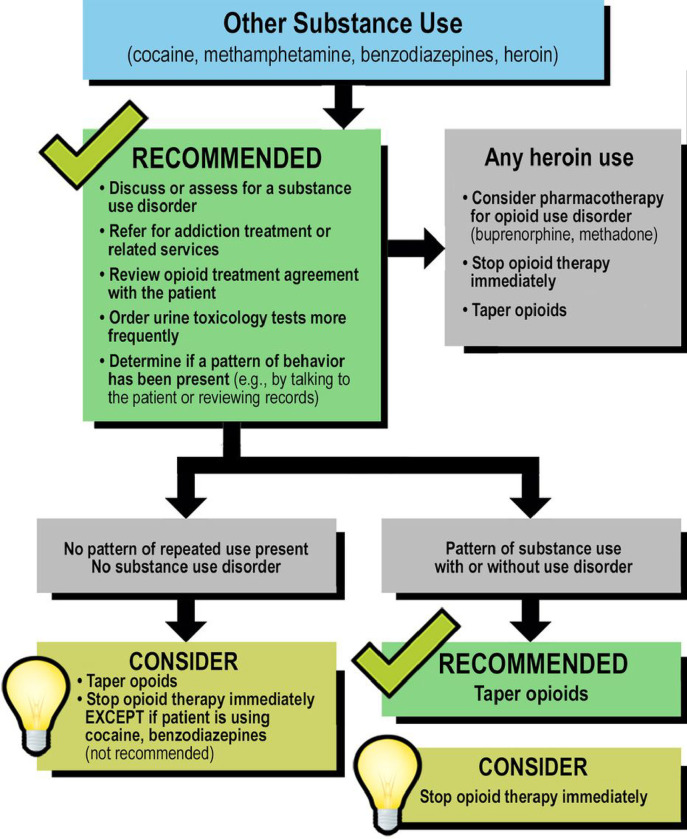
Legend not included with this version

We demonstrate the important and practical use of SP methodology for developing implementation strategies for a new EBP: 6 algorithms designed to address common and challenging behaviors associated with long-term opioid therapy (LTOT) developed by Merlin and colleagues.^14^ These algorithms were developed using Delphi methodology^15,16^ to find consensus on how to respond to behaviors such as missing appointments with clinicians prescribing the opioid, taking more opioid than prescribed, and substance use. One of the algorithms is included as an example in Fig. 1. We conducted SP sessions with providers using 6 cases, one for each algorithm. These SP sessions were followed by one-on-one structured interviews with questions mapping onto domains from the CFIR. [Figure 1: “Other Substance Abuse” Algorithm]

### 2.a Case development

We developed 6 SP cases. Each case simulated a patient exhibiting a unique concerning behavior addressed by the algorithms (see Table 1 outlining the behaviors). The cases were written with unfolding steps to represent three visits with a provider, because the algorithms guide decision points that would normally occur in subsequent follow-up visits in real-life practice (Fig. 1). The unfolding nature of the scenarios was piloted early in the case development process to ensure feasibility.

Cases were next reviewed by a Patient-Provider Advisory Board (PPAB) consisting of 3 patients with lived experience with opioids, 4 researchers (among whom are PCPs familiar with caring for patients with opioid misuse disorder), and a primary care provider with familiarity with providing care for patients with opioid misuse. Cases were edited based on feedback from the PPAB. In concert with the review of the 6 cases, the PPAB reviewed the instructions which provided context, expectations for patient interactions, and training on the algorithms (See Appendix). Finally, cases and instructions were piloted with an SP and a provider outside of the panel. During this pilot, a physician with topical expertise was recruited to interact with SPs portraying two cases over three subsequent visits on a remote platform. This pilot helped to further develop the other five cases in structuring how participants would be oriented, updated, and guided through the simulations.

### 2.b SP training

SPs in the University of Pittsburgh School of Medicine SP Program receive foundational training in case portrayal, providing feedback, supported physical exam training, and checklist scoring. This 16-hour onboarding combines both active training and also guided observation of SP activities. It prepares SPs to identify, recognize, and reward learner skill in portrayal, and to record it faithfully in assessments.

Four experienced SPs were recruited from the University of Pittsburgh SP program to portray the patients exhibiting misuse behaviors. To allow rotation, redundancy and information sharing, the SPs worked in pairs for each case, alternating the role of moderator and patient. When not portraying the patient, the SP acted as a moderator by providing clinicians with inter-visit updates in accordance with what the clinicians ordered in the first session and noted the passage of time between visits. A fifth experienced SP was recruited to proctor the event – orienting the participants and researchers as they arrived, running the Zoom sessions, and serving as a backup should one of the other SPs not be able to participate. They also were given an overview of case content, portrayal, and event structure. SPs were provided with case materials a week in advance of the portrayal date, were able to ask questions over email, and completed a case-specific training to align portrayal with parameters provided in the inter-visit updates with SP staff in the 45 minutes preceding the simulation. The SP program follows the Association for Standardized Patient Educators Standards of Best Practice, which “were written to ensure the growth, integrity, and safe application of SP-based education practices.”^9^

### 2.c Description of session for participants

Clinical participants were emailed information and instructions about the event prior to participating in the session (see Appendix). All sessions were held virtually via the Zoom interface due to the COVID pandemic. During the sessions, there was a brief orientation for participants. The orientation included: (1) A brief training in how to use the algorithms; (2) An overview of how to approach the simulated interaction (i.e., as close to real practice as possible); and (3) An overview of the one-on-one interview that would follow to discuss the approaches to implement the management algorithms.

Participants then moved into Zoom breakout rooms to begin their patient encounters. Participants were given up to 60 minutes to have their 3 distinct visits per patient. There was a 15-minute break, and then another 60 minutes for the second patient scenario.

For each of the 60-minute SP scenarios, participants were told that they were about to see a patient who was being seen by one of their partners (Dr. Williams) who recently left the practice. Dr. Williams had started the patient on opioid therapy and had an opioid agreement with the patient. Participants were given a copy of Dr. Williams’ last progress note and the opioid agreement prior to meeting the patient. After reviewing this information, the clinicians joined a Zoom breakout room with the SP portraying their patient. Once the provider ended the first encounter, the portraying SP turned off their camera, and, to reflect the passage of time between visits, the moderator gave the clinicians the results of any testing they ordered and any information about the patient that had changed between the last and next visit. The provider indicated when they were ready to start the next encounter. This process was repeated between the second and third encounter.

### 2.d Data Collection: Semi-Structured Interviews

Immediately after they interacted with the SPs, each participant completed a one-on-one interview to reflect on and assess the experience, as well as to provide feedback on how the algorithms should ultimately be integrated into practices like theirs. Interviews were conducted by experienced qualitative data specialists associated with Qualitative, Evaluation and Stakeholder Engagement Research Services (Qual EASE) at the University of Pittsburgh. Interviewers used a semi-structured interview guide developed by the research team that covered the following domains: (1) Assessment of their orientation to the algorithms, including training; (2) Assessment of their interaction with the SPs; (3) Assessment of and opinions on the algorithms; and (4) Description of how they thought the algorithms would operate in their practices, and how they could best be implemented there. Interviews were conducted on Zoom and recorded.

Questions and further probing were used to best assess how the algorithms could be implemented in their practices, which map onto several CFIR domains and constructs as shown in Table 2.

Within one week of their completion, the qualitative methodologist associated with the project wrote a summary of each interview, which was forwarded to the study team so that they could begin to determine what modifications might need to be made to the algorithms and could plan for implementation. Following that initial summary, interviews were transcribed verbatim with identifying details redacted. Under the supervision of the qualitative methodologist, experienced analysts at Qual EASE inductively developed a codebook reflecting the content of the interviews, with coding categories reflecting the four areas of the interview guide mentioned above. Use of the codebook was practiced on two transcripts by 2 Qual EASE coders, following which they both applied the codebook to the remaining 10 transcripts. Cohen’s Kappa statistics were used to assess intercoder reliability; the average kappa score was 0.8565, indicating “near perfect” agreement. The primary coder for the project then conducted a content and thematic analysis, which was reviewed by the qualitative methodologist, and shared with the study team to better facilitate implementation planning.

### 2.e Data Collection: Development of Implementation Bundle

The final step to developing the implementation bundle – which included materials for initial training, an online algorithm interface, e-consultation support, and electronic health record (EHR) integration for the 6 algorithms – was to review notes from the structured interviews. The bundle was then drafted and reviewed by the PPABs and co-Is.

### 2.f Recruitment and Study sample

Recruitment emails were sent to Community Medical Inc. (CMI). CMI is a network of 400 primary care and specialty physicians who practice throughout western and central Pennsylvania and provide care for over 495,000 patients. The practices cover a large geographic area; however, the network is predominantly in Allegheny County. Participants were required to be primary care clinicians at CMI practices and at least 18 years of age. Each of the clinicians were recruited to participate in two virtual patient evaluations followed by one-on-one interviews. The experience lasted approximately four hours and clinicians were paid $1,000 for their participation. We ultimately recruited 12 PCPs to participate in the virtual experience.

## Results

### 3.a Implementation support strategies

When asked about how algorithms should be implemented in practices like theirs, clinicians indicated that the orientation they had received to the algorithms would be a useful implementation support strategy. Other themes illustrating helpful implementation support strategies included: (1) The importance of having the algorithm use endorsed by practice leadership, and of having a local “champion” who promoted their use; (2) Integration of the algorithm workflow into practice EHRs; (3) Practice and location-specific inputs into the algorithms, such that a suggestion to refer to a specialist come with a list of who, specifically, to refer to, or a suggestion to call security provide the practice-specific number for security; (4) Access to specialists who could help interpret unclear or difficult-to read drug screens or suggest a particular course of action with a tricky patient.

Representative quotes supporting these themes, as well as the CFIR domains that they map to, are provided in Table 3. These findings were integrated into an implementation toolkit that included an initial training session followed by a suite of supports, including EHR integration, algorithm guidance hosted on a separate website with links to useful tools, and support for clinicians via e-consultation.

### 3.b Simulation Feedback from Clinicians

We identified two themes related to the physicians’ encounters with the SPs: (1) Clinicians found it useful to practice the algorithms with the SPs; (2) While clinicians applauded the skill of the SPs, they noted that not all actual patient counters go so smoothly. Each is presented in more detail below.

#### 3.b.i Clinicians found it useful to practice the algorithms with the SPs

Clinicians interviewed found it useful to practice the algorithms with the SPs. As will be discussed below, not all clinicians found the scenarios or SP reactions to be fully realistic. However, they did find practicing the algorithms in this way to be a useful way of learning the algorithms. As one provider put it:

It was a good chance to sort of get to look through the algorithm while I’m talking to them and sort of follow along. So, that was good to get familiar with the algorithm itself in a situation where you don’t feel like you’re with a real patient who you’re, like, ignoring to read through the algorithm.

Another provider similarly reflected:

So, that was really helpful, because this is sort of cut and dry of the way it’s written. And not until you’re in an actual patient scenario do you see some of the gray nuances. For example, one of the cases, the patient was having trouble sleeping secondary to pain. So, she was using her oxycodone in the evening to help with sleep, but it was related to pain. So, it wasn’t this clear-cut ‘I’m just using this to fall asleep at night.’ It was ‘I’m using this because at night my pain is worse which is affecting my sleep, so that’s why I’m using it.’ Which is a gray space. So, having the algorithm to sort of follow through and use as a guide let me make sure I’m asking all the right questions, let me make sure I’m offering all the other alternative things, was definitely beneficial.

#### 3.b.ii While clinicians applauded the skill of the SPs, they noted that not all actual patient counters go so smoothly

Many clinicians described the practice session with SPs as being realistic or very similar to encounters with real patients. One provider described themselves as “shocked” at how realistic the SPs were, adding that *“I felt very engaged in each of the scenarios. Like, they knew their background, they kind of were living the patient. I was really impressed... the scenarios were spot-on.”* Other clinicians described the scenarios as *“realistic situations that you can see in the office every day,” and “totally realistic.”*

However, some clinicians described pointed differences with real life patient visits. For example, the following provider described that some of their actual patients would simply never agree to the treatment plans presented in the algorithms:

In the back of my mind I’m thinking of my actual patients who I’ve run into these instances and how this would go, and I don’t think it would’ve – it won’t go the way that it went with the SPs. Because it sometimes doesn’t matter how good your rapport is, they just aren’t gonna do what’s suggested... I think I run into much harder stops with some of my real non-SP patients.

Another clinician echoed this description, noting that:

My experience is that patients don’t normally accept what you say so easily. […] The interactions that I have with my patients are not anything like these, ‘cause these were very calm, very reasonable, willing to listen to you; they seemed to have a health literacy level that is well beyond a lot of the patients I deal with.

While these concerns were not voiced by every participant, they were voiced by participants who experienced different scenarios with the SPs, indicating that patients may not always be agreeable to the actions suggested in the algorithm – and that that lack of agreement would be something that would need to be managed in an ongoing patient relationship, rather than disappearing at the end of the role play with the SP.

## Discussion

In this study, we used the SP methodology in combination with one-on-one interviews guided by CFIR to develop an implementation bundle for 6 algorithms designed to address common and challenging behaviors associated with LTOT. We found the use of the SP methodology to be a valuable tool for highlighting important components of an implementation bundle. Specifically, we found that an implementation bundle addressing (1) The importance of having the algorithm use endorsed by practice leadership, and of having a local “champion” who promoted their use; (2) Integration of the algorithm workflow into practice EHRs; (3) Practice and location-specific inputs into the algorithms would be most effective in promoting the successful adoption and implementation of the EPBs for the LTOT algorithms. We also found that the SPs were realistic in their portray of patients with LTOT; however, it was noted that patients of the clinicians that participated in the simulations were likely to be more resistant to the adoption of the recommendations outlined by the algorithms than the SP portrayal. SPs are trained to recognize and reward participant skill, which may account for this observation.

Of methodological note in the realm of qualitative research: completing the interviews just after the SP interactions set an excellent stage for collecting qualitative data, likely because clinicians had just had a novel experience that was fresh in their minds. They could also talk about the details of the SP cases without concern for inappropriately describing actual patient cases in too much detail and contrast the SPs with their patients in general. This made for highly engaging interviews in which rapport building between interviewer and interviewee was more easily built. Additionally, interviews were conducted by qualitative research specialists who were not personally invested in the development of the algorithms or orientation to the algorithms, setting the stage for open and honest feedback.

## Conclusions

Overall, this study demonstrates the potential of using the SP methodology guided by the CFIR framework to develop effective implementation strategies for improving care in real-world healthcare settings. The use of SPs allowed the research team to observe the EBP in practice with feedback from end-users with experience from different health care clinics. The CFIR framework provided a comprehensive approach to guiding the development of an implementation bundle that addressed the multiple factors that influence EBP implementation.

## Figures and Tables

**Table 1:** SP Cases

Case	Algorithm
1	Missing Appointments
2	Taking Opioids for Symptoms Other Than Pain
3	Using More Opioid Medication Than Prescribed
4	Asking for an Increase in Opioid Dose
5	Alcohol Use
6	Other Substance Use

The algorithms created by Merlin, Young, Azari, et al. 2016 also addressed “Aggressive Behavior”; however, based on feedback from the patient-provider advisory board, we did not review the algorithm in the present study.

**Table 2:** Mapping of CFIR domains and constructs in interview guide

CFIR Domain (definition in parenthesis)	Selected Constructs from Domain	Corresponding Question(s)
Innovation (Characteristics of “the thing” being implemented, i.e., the innovation)	Innovation Design	What is your general impression of the algorithms? Probes: What, if anything, about them is useful? What, if anything, about them was unhelpful?
	Innovation Adaptability	How would the description (or orientation) of the algorithms that we presented need to be tailored to your practice? What, if any, edits did you find yourself wanting to make to the algorithms?
Outer setting (The setting in which the inner setting exists, e.g., the health system and community in which the practice sits)	Local Conditions	What clinic (or health system) level factors would make implementing these algorithms easy? What factors would make it difficult?
	Partnerships and Settings	How, if at all, would insurance company policies impact your ability to implement these algorithms?
Inner setting (The setting in which the innovation will be implemented, e.g., the individual practice)	Work Infrastructure	How would you fit the algorithms into your workflow at your practice? How would you like to use these algorithms in your day-to-day practice (if you would like to use them – if you wouldn’t like to use them, we’d love to hear about that too)?
	Relational Connections	Next, I would like to talk about implementation of these algorithms in practices like yours. First, tell me about your practice. Probes: How many providers are there? How many patients are in it? How would you describe your patient base?
	Information Technology Infrastructure	What would be the benefits/drawbacks of incorporating the algorithms into the EHR? How would you do it?
Individuals (The roles and characteristics of individuals involved in or affected by implementation of the innovation)	Innovation Recipients	How do you think patients will respond to these algorithms, and what types of things do clinicians need to do make this acceptable to patients? What would make it easy or difficult to implement these algorithms with a given patient?
	High-Level Leaders	How could we best get practice and PCP buy-in to implement these algorithms?
	Mid-Level Leaders	What would make it easy or difficult for individual PCPs to implement these algorithms?
Implementation process (The activities and strategies used to implement the innovation)	Tailoring Strategies	This morning, we provided you with a brief orientation (or “introduction”), or description of the algorithms. What was good about the initial description? What could have been better? How would the description (or orientation) of the algorithms that we presented need to be tailored to your practice? How would you want doctors in your practice to be oriented [trained] to the algorithms? If these algorithms were implemented in your practice, what type of ongoing training, if any, would you and your colleagues want to receive? What kind of interactive assistance would be helpful? (Having a support person in clinic? Over the phone?) What kind of patient-level support would be helpful? (i.e., What kind of supports do you need for your patients in order for you to use the algorithms effectively?) (Infrastructure? Financial?)

**Table 3:** Quotes from Semi-Structured Interviews

Theme	CFIR Domain	Quotes
The importance of having the algorithm use endorsed by practice leadership, and of having a local “champion” who promoted their use.	*Innovation, Outer Setting, Inner Setting, Individuals*	*“If it comes from administration, from the top, you think it’s more validated. ... if it just comes from a pain practice, it’s less likely, because we don’t know if it just comes from their opinion ... if it’s been implemented at a specific practice already and has been useful and believed to be efficient, and is easily implementable, ... if somebody who does this and tells you, yes, this easily implemented, even despite all the things we have to do, this does help me make things faster, um, then it’s more likely for other people to look at it. (Participant 11)*
		*“If you got the head of all the private practice providers to say this is our-what we’re gonna do, ... at least everyone would have the same set of tools, ... instead of sort of saying here’s a bunch of things, you know, do in your office how you will. ... while that gives flexibility, it also allows for a lot of variability. ... if there’s way too much variability, then, you know – like that last standardized patient said to me, I’ll just switch doctors. ... because they don’t like the answer that they’re given. So, if you knew that it was gonna be consistent, then maybe people would be a little less apt to do that.” (Participant 2)*
Integration of the algorithm workflow into practice EHRs.	*Inner Setting*	*“If it becomes a smart set or an EPIC list or something like that where you have a check-off, if it’s a missed appointment issue and it goes into that segment of the algorithm versus the other pieces, of other substance use or taking opioids for symptoms other than pain and those kinds of things. I think having kind of a check-off box that leads you to the next [step] – and then it opens kind of the next step piece would be helpful.” (Participant 4)*
		*“I think converting these to templates in EPIC that we can instantly import to them into the chart. Because, no matter how good we are, these papers are going to get lost in a huge, massive pile of papers in the office. So, if I can ... just enter dot I G opiates, early appointment, early prescription, and gives me the questionnaire and-and I go over the questions, we’re good. ... Because our office hours are extremely, extremely tight. Every day there are new things that we need to add doing to our patients. While you are talking to them, talk about colon cancer screening. Um, you can easily spend half an hour convincing someone to take the COVID vaccine and... So, just-just so add-ons. So, if this is going to be a part of usually many other things in that visit, we need to be very time-efficient. (Participant 5)*
Practice and location-specific inputs into the algorithms, such that a suggestion to refer to a specialist come with a list of who, specifically, to refer to, or a suggestion to call security provide the practice-specific number for security.	*Inner Setting, Implementation Process*	*“It would need to have some specific numbers. So, things like call security. ... So, it’s not like I have to go look something up while I’m dealing with someone with an aggressive behavior, right? [and] when it says refer to specialist ... an addiction specialist number, chronic pain number, those listed out so, again, we’re not having to dig for them.” (Participant 3)*
		*“[We need direct referrals or embedded practitioners because] if you’re going through the algorithm and it says, you know, address underlying problems, and their underlying problem is uncontrolled anxiety, that’s what they need is therapy, and you don’t have it. ... And telling people to call the back of their card to figure out who takes their insurance is, like, archaic at this point.” (Participant 1)*
Access to specialists who could help interpret unclear or difficult-to read drug screens, or suggest a particular course of action with a tricky patient.	*Outer Setting, Implementation Process*	*“It would be helpful to have better pain management resources, especially in terms of medication management. Somebody that I can reach out to and say, ‘hey, listen, can you eyeball the chart. ...what do you think?’ Like, [an] opioid management e-consult. They have that paradigm already for some other health conditions.” (Participant 12)*
		*“I think another potential place could be if the consultants that were calling for help saw a pattern of, like, they keep asking the same question that I keep answering, that would be a time to be like, maybe we need to re-educate this piece, because I keep getting the same call from different people. So, those-those folks might help steer directions of, like, where our-where there’s an education lapse.” (Participant 3)*

## Data Availability

The dataset supporting the conclusions of this article is available from the corresponding author on reasonable request.

## Abbreviations

CFIR: Consolidated Framework for Implementation Research
CMI: Community Medical Inc
Co-I: Co-Investigator
EBP: Evidence-based practice
EHR: Electronic health record
LTOT: Long-term opioid therapy
PCP: Primary care physician
PPAB: Patient-Provider Advisory Board
SP: Simulated patient

## References

[1] Luciano M, Aloia T, Brett J. 4 Ways to make evidence-based practice the norm in Health Care. Harvard Business Review. 2019;4

[2] Rapport F, Clay-Williams R, Churruca K, Shih P, Hogden A, Braithwaite J. The struggle of translating science into action: Foundational concepts of implementation science. J Eval Clin Pract. Feb 2018;24(1):117–126. doi:10.1111/jep.12741 28371050 PMC5901403

[3] Brownson RC, Jacobs JA, Tabak RG, Hoehner CM, Stamatakis KA. Designing for dissemination among public health researchers: findings from a national survey in the United States. Am J Public Health. Sep 2013;103(9):1693–9. doi:10.2105/AJPH.2012.301165 23865659 PMC3966680

[4] Powell BJ, Waltz TJ, Chinman MJ, A refined compilation of implementation strategies: results from the Expert Recommendations for Implementing Change (ERIC) project. Implementation science. 2015;10(1):1–14. 25889199 10.1186/s13012-015-0209-1 PMC4328074

[5] Barrows HS. An overview of the uses of standardized patients for teaching and evaluating clinical skills. AAMC. Academic Medicine. 1993;68(6):443–51. 8507309 10.1097/00001888-199306000-00002

[6] Liao C-S, Hsieh M-C. Standardized Patient Training: Using ANGER to quickly evoke anger in standardized patients. Medical Teacher. 2015;37(9):883–883. 10.3109/0142159X.2014.993956 25523116

[7] Zabar S, Hanley K, Kachur E, “Oh! She doesn’t speak English!” Assessing resident competence in managing linguistic and cultural barriers. Journal of general internal medicine. 2006;21:510–513. 16704400 10.1111/j.1525-1497.2006.00439.x PMC1484779

[8] Taylor LJ, Adkins S, Hoel AW, Using Implementation Science to Adapt a Training Program to Assist Surgeons with High-Stakes Communication. J Surg Educ. 2019;76(1):165–173. doi:10.1016/j.jsurg.2018.05.015 30626527

[9] Bryant KA, Wesley GC, Wood JA, Hines C, Marshall GS. Use of standardized patients to examine physicians’ communication strategies when addressing vaccine refusal: a pilot study. Vaccine. Jun 02 2009;27(27):3616–9. doi:10.1016/j.vaccine.2009.03.048 19464542

[10] Lewis KL, Bohnert CA, Gammon WL, The association of standardized patient educators (ASPE) standards of best practice (SOBP). Advances in Simulation. 2017;2(1):1–8. 29450011 10.1186/s41077-017-0043-4 PMC5806371

[11] Wilson L, CHSE C-A, Wittmann-Price RA. Review manual for the certified healthcare simulation educator exam. Springer Publishing Company; 2018.

[12] Jeffries P. Clinical simulations in nursing education: Advanced concepts, trends, and opportunities. Lippincott Williams & Wilkins; 2022.

[13] Breimaier HE, Heckemann B, Halfens RJ, Lohrmann C. The Consolidated Framework for Implementation Research (CFIR): a useful theoretical framework for guiding and evaluating a guideline implementation process in a hospital-based nursing practice. BMC nursing. 2015;14:1–9. 26269693 10.1186/s12912-015-0088-4 PMC4533946

[14] Merlin JS, Young SR, Azari S, Management of problematic behaviours among individuals on long-term opioid therapy: protocol for a Delphi study. BMJ open. 2016;6(5):e011619. 10.1136/bmjopen-2016-011619 PMC4861114 27154486

[15] Dalkey N, Helmer O. An experimental application of the Delphi method to the use of experts. Management science. 1963;9(3):458–467.

[16] Keeney S, McKenna HA, Hasson F. The Delphi technique in nursing and health research. John Wiley & Sons; 2011.

[17] Damschroder LJ, Aron DC, Keith RE, Kirsh SR, Alexander JA, Lowery JC. Fostering implementation of health services research findings into practice: a consolidated framework for advancing implementation science. Implementation science. 2009;4(1):1–15. 19664226 10.1186/1748-5908-4-50 PMC2736161

